# Pharmacogenetics of dolutegravir plasma exposure among Southern Africans living with HIV

**DOI:** 10.1093/infdis/jiac174

**Published:** 2022-05-04

**Authors:** Zinhle Cindi, Aida N. Kawuma, Gary Maartens, Yuki Bradford, Francois Venter, Simiso Sokhela, Nomathemba Chandiwana, Roeland E. Wasmann, Paolo Denti, Lubbe Wiesner, Marylyn D. Ritchie, David W. Haas, Phumla Sinxadi

**Affiliations:** 1 Division of Clinical Pharmacology, Department of Medicine, University of Cape Town, Cape Town, South Africa; 2 Wellcome Centre for Infectious Diseases Research in Africa, Institute of Infectious Disease and Molecular Medicine, University of Cape Town, Cape Town; 3 Department of Genetics, University of Pennsylvania, Philadelphia, PA, USA; 4 Ezintsha, Faculty of Health Sciences, University of the Witwatersrand, Johannesburg, South Africa; 5 Genomics and Computational Biology Program, University of Pennsylvania, Philadelphia, Pennsylvania, Department of Genetics, University of Pennsylvania, Institute for Biomedical Informatics, University of Pennsylvania, Philadelphia, Pennsylvania, USA; 6 Department of Medicine, Vanderbilt University Medical Center, Nashville, Tennessee, USA; 7 Department of Internal Medicine, Meharry Medical College, Nashville, Tennessee, USA

**Keywords:** Antiretroviral therapy, dolutegravir, HIV, pharmacogenetics, pharmacokinetics

## Abstract

**Background:**

Dolutegravir is a component of preferred antiretroviral therapy (ART) regimens. We characterised the pharmacogenetics of dolutegravir exposure following ART initiation in the ADVANCE trial in South Africa.

**Methods:**

Genome-wide genotyping followed by imputation was performed. We developed a population pharmacokinetic model for dolutegravir using non-linear mixed-effects modelling. Linear regression models examined associations with unexplained variability in dolutegravir area under the concentration-time curve (AUC_VAR_).

**Results:**

Genetic associations were evaluable in 284 individuals. Of nine polymorphisms previously associated with dolutegravir pharmacokinetics, the lowest P-value with AUC_VAR_ was *UGT1A1* rs887829 (P = 1.8 x 10^-4^), which was also associated with log_10_ bilirubin (P = 8.6 x 10^-13^). After adjusting for rs887829, AUC_var_ was independently associated with rs28899168 in the *UGT1A* locus (P = 0.02), as were bilirubin concentrations (P = 7.7 x 10^-8^). In the population pharmacokinetic model, rs887829 T/T and C/T were associated with 25.9% and 10.8% decreases in dolutegravir clearance, respectively, compared to C/C. The lowest P-value for AUC_VAR_ genome-wide was *CAMKMT* rs343942 (P = 2.4 x 10^-7^).

**Conclusions:**

In South Africa, rs887829 and rs28899168 in the *UGT1A* locus were independently associated with dolutegravir AUC_VAR_. The novel rs28899168 association warrants replication. This study enhances understanding of dolutegravir pharmacogenetics in Africa.

## Introduction

Sub-Saharan Africa has the highest prevalence of HIV-1 infection worldwide (1). Dolutegravir is an HIV-1 integrase strand transfer inhibitor with a favourable safety profile and high barrier to viral resistance (2). The World Health Organization recommends dolutegravir with tenofovir and lamivudine as a preferred regimen for ART-naïve patients, and dolutegravir-based ART as a switch option when failing ART or transitioning for programmatic reasons (3). In South Africa, preferred initial regimens include dolutegravir, together with either tenofovir and emtricitabine, or abacavir with lamivudine (4).

Dolutegravir undergoes glucuronidation by hepatic uridine glucuronosyltransferase 1A1 (UGT1A1), with minimal contributions of cytochrome P450 (CYP) 3A4, UGT1A3 and UGT1A9 (5), and is a substrate of transporter proteins P-glycoprotein (encoded by *ABCB1*) and breast cancer resistance protein (encoded by *ABCG2*) (6).

Studies of ART pharmacogenetics may guide ART optimization in different populations. Africa has the world's greatest genetic diversity (7), but data are limited regarding dolutegravir pharmacogenetics in Africa. We characterized genetic associations with dolutegravir exposure among ART-naïve African participants in the ADVANCE study (8).

## Methods

### Study population

The ADVANCE study in South Africa was a phase 3 clinical trial (clinicaltrial.gov NCT03122262) in which 1,053 HIV-positive, ART-naïve participants were randomly assigned to one of three treatment arms: 1) dolutegravir, tenofovir alafenamide (TAF) and emtricitabine; 2) dolutegravir, tenofovir disoproxil fumarate (TDF) and emtricitabine; or 3) efavirenz, TDF and emtricitabine (8). The present study included dolutegravir arm participants who consented to genetic testing.

### Pharmacokinetic sampling and analysis

Intense pharmacokinetic sampling at steady state was performed in a subset of participants receiving dolutegravir, equally divided between TDF- and TAF-containing arms. Samples were taken pre-dose and at 1, 2, 4, 6, 8, and 24 hours post-dose. Doses preceding intense sampling were observed following a standard meal. For all other individuals, sparse pharmacokinetic sampling (at least one sample) was performed at either week 48 or 96.

Dolutegravir was quantified with a validated assay developed at the Division of Clinical Pharmacology, University of Cape Town. Samples were processed with a liquid-liquid extraction method using dolutegravir-d4 as an internal standard, followed by high performance liquid chromatography with tandem mass spectrometry detection (LC/MS/MS) using an AB SCIEX API 4000 instrument. Analyte and internal standard were monitored at mass transitions of the protonated precursor ions m/z 420.1 and m/z 424.2 to the product ions m/z 277.2 and m/z 279.1, respectively. The calibration curve fitted a quadratic regression over the range 0.030 to 10.0 μg/mL. Combined accuracy and precision statistics of quality control samples during validation were between 103.5% and 106.0%, and 4.6% and 6.1%, respectively. The laboratory participated in the Clinical Pharmacology Quality Assurance external quality control program under a contract with the Division of AIDS of the National Institute of Allergy and Infectious Diseases, through which this assay was approved.

### Genetic polymorphisms

Whole blood was collected from consenting participants, and DNA extracted as described elsewhere (9). Samples were labelled with coded identifiers. Stored DNA was genotyped using the Illumina Infinium Multi-Ethnic Global BeadChip (MEGA^EX^) at Vanderbilt Technologies for Advanced Genomics (VANTAGE). Post-genotype quality control included sex checks, call rates by marker and sample, identity by descent (IDB) plots, assessment for batch effects, concordance between duplicate samples, and HapMap controls.

Quality control steps were performed using PLINK version 1.9 (10). Genotyping efficiency per participant was > 95% for all samples. After quality control, data were imputed using the TOPMed reference panel after transforming to genome build 38 using liftOver and stratification by chromosome to parallelize the imputation process (11). We excluded imputed polymorphisms with imputation scores < 0.3, genotyping call rates < 95%, minor allele frequency (MAF) < 0.05, or Hardy-Weinberg Equilibrium P-values < 1.0 x10^-8^. Linkage disequilibrium (LD) *r*^2^ values were determined using PLINK.

### Population pharmacokinetic modelling (without genetics)

Concentration-time data were analysed by non-linear mixed-effects modelling with NONMEM (v7.5.0, ICON Development Solutions, Ellicott City, MD, USA). First-order conditional estimation with eta-epsilon interaction were used to fit models to the observations. Pearl-speaks-NONMEM (PsN) v4.7.0, Pirana v2.9.7, and R v3.6.1 were used for model automation and tracking, and to create and visualize model diagnostics. We tested various structural models to describe the pharmacokinetics of dolutegravir including one- and two-compartment disposition models, first-order elimination and absorption, with or without absorption lag time, and transit compartments (12). We included between-individual and between-occasion random effects on the model parameters with an assumption of a log-normal distribution. A combined (additive and proportional) error model was used to describe the residual unexplained variability, with the additive component of the error constrained to be at least 20% of the lower limit of quantification.

Throughout the pharmacokinetic analysis, model development was guided by inspection of diagnostic plots, including visual predictive checks (VPC), and decreases in the NONMEM objective function value (OFV), which were assumed to follow a chi-square distribution. To discriminate between nested models, a decrease in OFV of -3.84 was equivalent to model improvement at P < 0.05. To adjust for the effect of body size on disposition parameters, we added allometric scaling to the model. We tested total body weight and fat-free mass (FFM) with the allometric exponents for clearance and volume parameters fixed to 0.75 and 1, respectively (13). After the inclusion of allometric scaling, we investigated effects of the following covariates: sex, age, and TAF- versus TDF-containing ART. Covariates were assessed by stepwise inclusion followed by backward elimination and were retained at a significance level of 0.01. To evaluate the robustness of parameter estimates in the final model, we ran a sampling-importance resampling exercise and generated 95% confidence intervals (CI).

### Unexplained variability to be regressed against genetics

The final model was used to generate individual steady-state estimates of area under the concentration–time curve (AUC_0-24h_) and unexplained variability in AUC_0-24h_ (AUC_VAR_) for individuals with at least one pharmacokinetic sample. Individual estimates of all model parameters were obtained from the final model by a post-hoc Bayes estimation method, considering an individual's pharmacokinetic data and characteristics (i.e., FFM). Individual estimates of AUC_0-24h_ were obtained using the formula AUC_0-24h_= *F*_i_ x Dose_i_/CL_i_, where Dose represents the actual dose given to each individual and CL_i_ and *F*_i_ represent individual estimates of clearance and bioavailability, respectively.

### Genetic association analyses

The outcome of primary interest was AUC_VAR_, and secondarily unexplained variability in dolutegravir clearance (CL_BSV_). Multivariable linear regression models characterised associations with genetic polymorphisms. To adjust for genetic ancestry, we estimated continuous axes of ancestry incorporating the intersection of common autosomal genotypes using EIGENSTRAT (14). Principal components scree plots were used to assess whether components included in analyses were informative; based on this, two principal components were sufficient. Other than principal components, additional covariates were not included in association analyses because they were accounted for in the population pharmacokinetic model. We report regression coefficients (b) for additive associations with polymorphisms, where positive b values indicate an association with increased AUC_VAR_. The Bonferroni method was used to determine significance thresholds, with 0.05 divided by the number of polymorphisms tested in targeted polymorphism and gene analyses, and P < 5.0 x10^–8^ for genome-wide analyses.

We a *priori* selected 12 polymorphisms previously reported to be associated with dolutegravir exposure (*ABCB1* rs1128503, rs2032582, rs1045642 and rs3842; *ABCG2* rs2231142 and rs2231137; *CYP3A4* rs35599367; *CYP3A5* rs776746 (*3 allele); *NR1I2* rs2472677 and rs1523130; and *UGT1A1* rs4148323 (*6 allele) and rs8175347 (*28 allele)). We also selected rs887829 which is in strong linkage with rs8175347. Beyond these polymorphisms, we then stepwise prioritized sets of polymorphisms to interrogate so as to decrease the burden of multiple testing. We reasoned that polymorphisms previously and strongly associated with at least one drug-related phenotype, or that have been significantly genome-wide associated with any trait, are most likely to be true associations. We used as references the Pharmacogenomics Knowledge Base (PharmGKB, accessed 13 July 2021) (15) and NHGRI-EBI GWAS Catalog (accessed 19 January 2021) (16). In PharmGKB, 173 polymorphisms were previously associated with at least one drug-related phenotype (pharmacokinetics, efficacy, or toxicity) with levels of evidence of 1 (preponderance of evidence shows an association, replicated in multiple cohorts, and preferably with strong effect size) or 2 (moderate evidence of association, replicated but some studies may not show statistical significance, or with small effect size). In the GWAS Catalog, 89,716 polymorphisms were previously associated with any trait at P < 5.0 x10^-8^ in at least one study.

We prioritized polymorphisms common to both PharmGKB and the GWAS Catalog, considering these to have the most robust evidence for true associations. We secondarily explored all polymorphisms from PharmGKB and the GWAS Catalog (based on criteria described above), and all polymorphisms in our imputed genome-wide genotype data.

As a positive control, we tested for associations between *UGT1A* locus polymorphisms and screening bilirubin concentrations. Bilirubin is conjugated by UGT1A1, and there are strong associations between *UGT1A1* polymorphisms (e.g., rs887829) and bilirubin concentrations (17).

### Population pharmacokinetic modelling with genetic information

Based on results of our genetic association analyses, we assessed effects of selected polymorphisms on dolutegravir clearance, on all available pharmacokinetic data (intensive and sparse samples). In addition to participants with genotype data, both intensive and sparse pharmacokinetic data was available from other ADVANCE participants. For such individuals we assigned a phenotype using mixture modelling as described elsewhere (18).

## Results

### Study population

**Figure 1:**
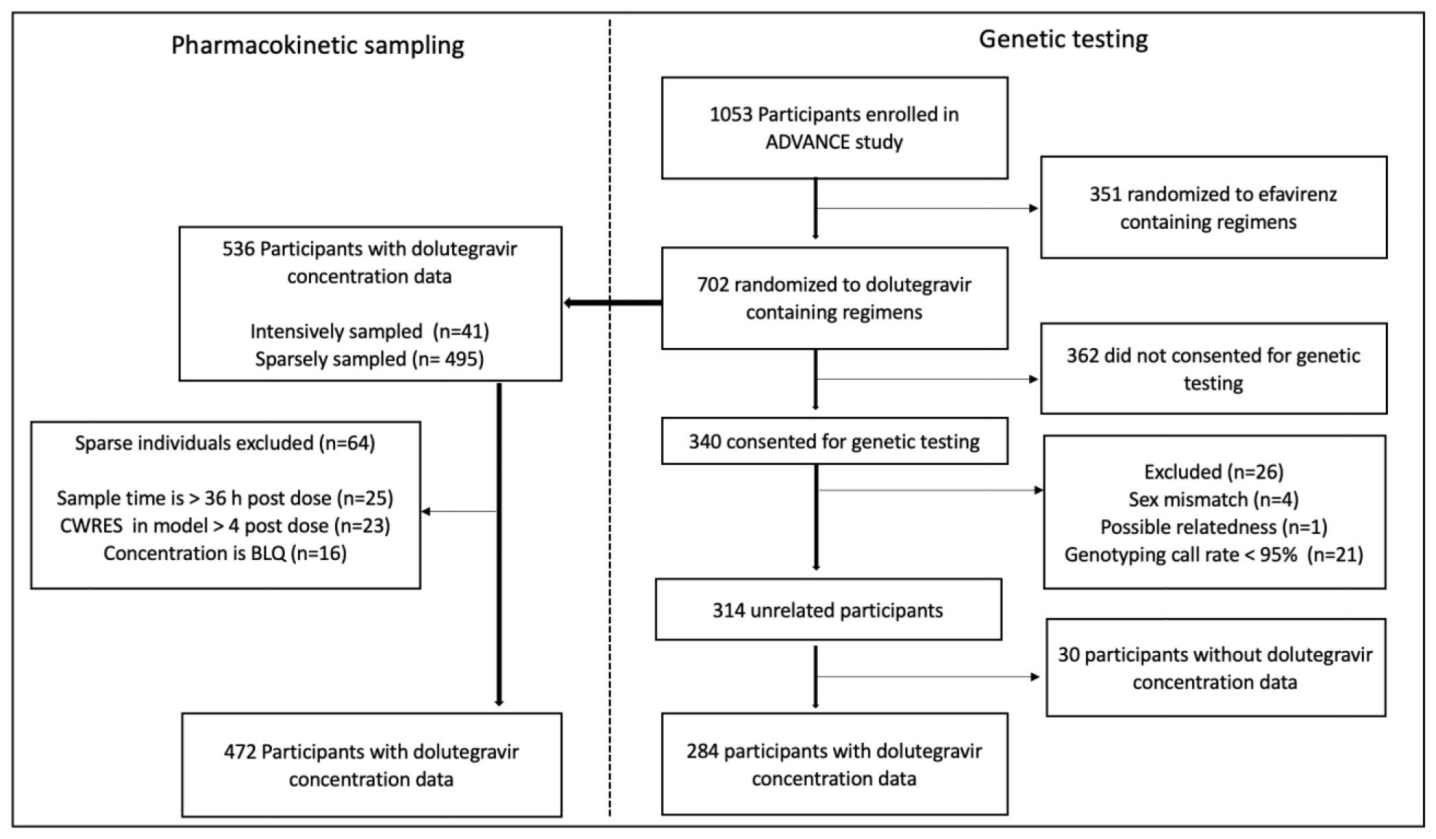
Disposition of study participants. Of 1,053 participants enrolled in the ADVANCE study, 284 who were randomized to dolutegravir-containing regimens and with available pharmacokinetic sampling data were evaluable for genetic associations. CWRES; conditional weighted residual, BLQ; below limit of quantification.

Among 340 ADVANCE participants who consented for genetic analyses, 284 (84%) were successfully genotyped and had pharmacokinetic data. Participant disposition is presented in Figure 1. All participants were Black Africans and 62% were females. Study participant characteristics are shown in Table 1.

### Dolutegravir population pharmacokinetics without genetics

For this model, data were available from 41 intensively sampled individuals who provided 276 dolutegravir concentrations (40 complete profiles, and 1 pre-dose sample from an individual for whom intravenous access difficulty precluded additional samples). Median (interquartile range) weight and age were 73.1 (67.2 – 85.2) kg and 31 (29 – 36) years, respectively. A two-compartment disposition model (ΔOFV=-47, P < 0.001 compared to one-compartment) with first-order elimination and transit compartments absorption (ΔOFV=-4.8, P = 0.028 compared to absorption lag) best described pharmacokinetics. Allometric scaling with FFM best described the effect of body size on disposition parameters and was applied to all clearance and volume parameters. We estimated clearance of 0.732 L/h, central volume of 12.2 L, and peripheral volume of 5.87 L for a typical individual with a 47 kg FFM and included between-subject variability on clearance and between-occasion variability on mean transit time (MTT), absorption rate constant, and bioavailability. Final parameter estimates, precision and the VPC shows that the model described the data adequately (Supplementary Data).

### Genetic associations with dolutegravir pharmacokinetics

Seven of 12 polymorphisms previously associated with dolutegravir pharmacokinetics were present in our genetic data. Of the five not included, three (rs4148323, rs8175347 and rs35599367) are very infrequent in African populations (MAF approximately 1% or less), and one was not genotyped (UGT1A1*28 rs8175347) but is in strong LD with rs887829 (19). Of the remaining seven polymorphisms, the lowest P-value for association with AUC_VAR_ was *UGT1A1* rs887829 (β = 0.14, P = 1.8 x 10^-4^) with a MAF of 0.41 (Table 2). This withstood correction for multiple testing (cut-off P < 5.6 x 10^-3^) and was also most strongly associated with CL_BSV_ (β = -0.09, P = 8.4 x 10^-6^) (Supplementary Data). Association of the other six polymorphisms with CL_BSV_ were consistent with those for AUC_VAR_ (data not shown). This was expected, since AUC is derived from CL (AUC_(0-24)_ = *F*_i_ x Dose_i_/CL_i_,) and these parameters were highly correlated (Spearman *r*^2^ = -0.96).

**Figure 2:**
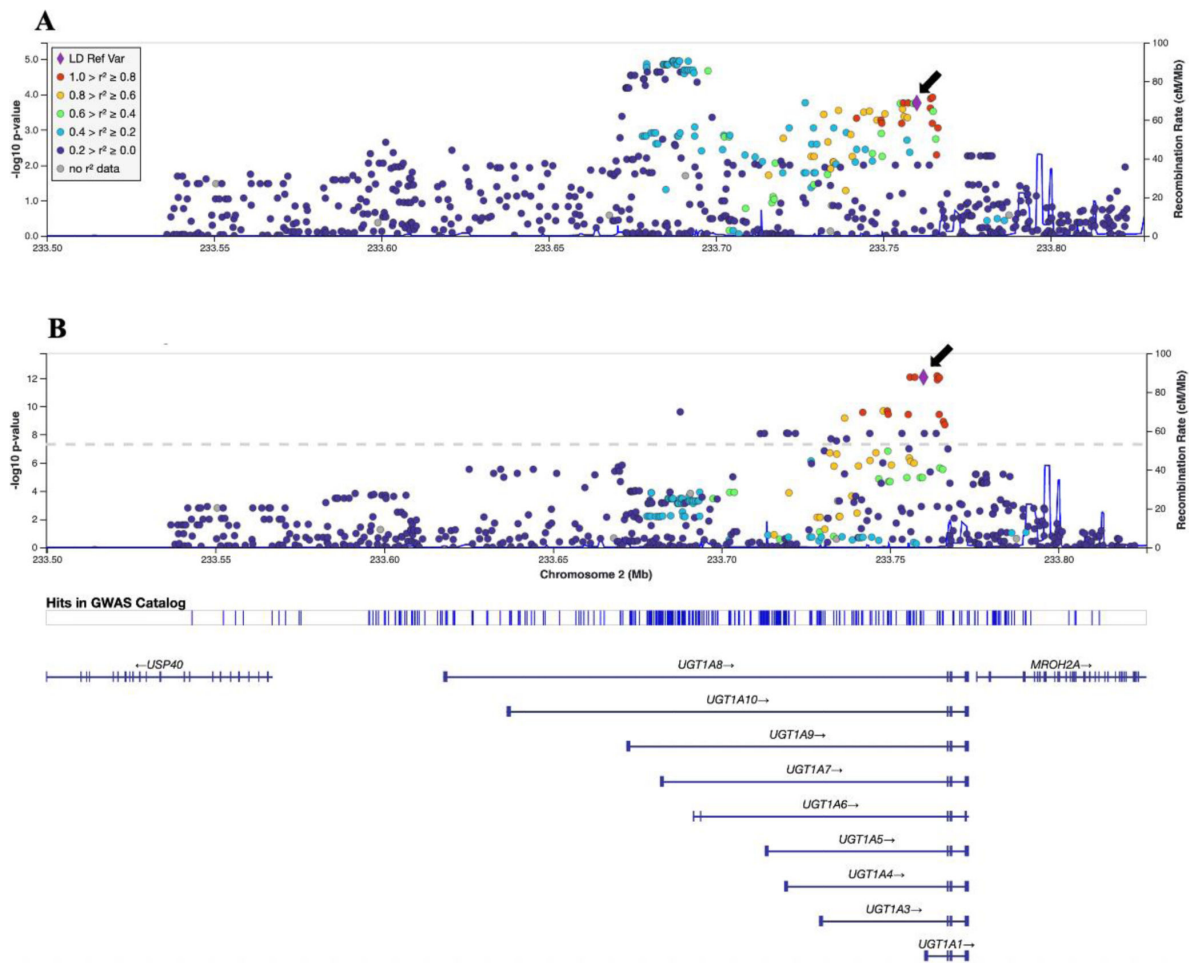
LocusZoom plots of *UGT1A* locus associations with plasma dolutegravir exposure and log_10_ bilirubin concentrations. The figure shows -log_10_ P-values for associations of 853 polymorphisms in the *UGT1A* locus (±50 kB in either direction) among 284 individuals evaluable for genetic associations. The purple diamond identifies *UGT1A1* rs887829 which we selected to be the reference polymorphism for linkage disequilibrium (LD) values because it has been most consistently associated with bilirubin concentrations in prior genome-wide association studies. Note the different Y-axis scales. *Panel A:* Associations with unexplained variability in dolutegravir AUC_VAR_. *Panel B:* Associations with baseline log_10_ bilirubin concentrations. Marker colors indicate LD *r*^2^ values in relation to rs887829, based on selecting the ALL populations option in LocusZoom.

To interrogate *UGT1A1* more thoroughly, we considered 853 polymorphisms within the *UGT1A* locus ± 50kb in each direction. The *UGT1A* locus includes *UGT1A1, 3, 4, 5, 6, 7, 8, 9* and *10* which create transcripts by differential splicing. A LocusZoom plot representing *UGT1A* locus associations is presented in Figure 2. For AUC_VAR_, the lowest P-value was rs6736508 (β = 0.18, P = 1.2 x 10^-5^) (Figure 2A), which withstood correction for multiple testing (cut-off P < 5.9 x 10^-5^). This polymorphism was not in strong LD with rs887829 (r^2^ = 0.32). By comparison, for log_10_ bilirubin concentrations the lowest P-value was rs6742078 (β = 0.11, P = 7.0 x 10^-13^), which was in strong linkage disequilibrium with rs887829 (Figure 2B). Results for CL_BSV_ were consistent with results for AUC_VAR_, although for CL_BSV_ no polymorphism had substantially lower P-values than rs887829. For CL_BSV_, the lowest P-value was rs201393786 (β = 0.1, P = 2.6 x 10^-7^) (Supplementary Data).

To determine whether rs6736508 or other *UGT1A* locus polymorphism were associated with AUC_VAR_ independent of rs887829, we repeated the above analyses after adjusting for rs887829, which should strengthen any true independent association. In this analysis, rs6736508 became 2-log less significant (β= 0.13, P = 4.2 x 10^-3^), and no longer withstand correction for multiple testing, indicating that its association was largely if not entirely dependent on rs887829. Results for CL_BSV_ were consistent with results for AUC_VAR_ (data not shown). To guide next steps, we used log_10_ bilirubin as a biomarker to identify additional variants that may affect UGT1A1 activity independent of rs887829. After adjusting for rs887829, a *UGT1A* locus polymorphism rs28899168 was independently associated with lower log_10_ bilirubin concentrations (β = -0.11, P = 7.7 x 10^-8^). This polymorphism was also independently associated with lower AUC_VAR_ (β = -0.12, P = 0.02). P-values for *UGT1A* locus polymorphisms with AUC_VAR_ and CL_BSV_ after adjusting for rs887829 are presented in Supplementary Data.

**Figure 3:**
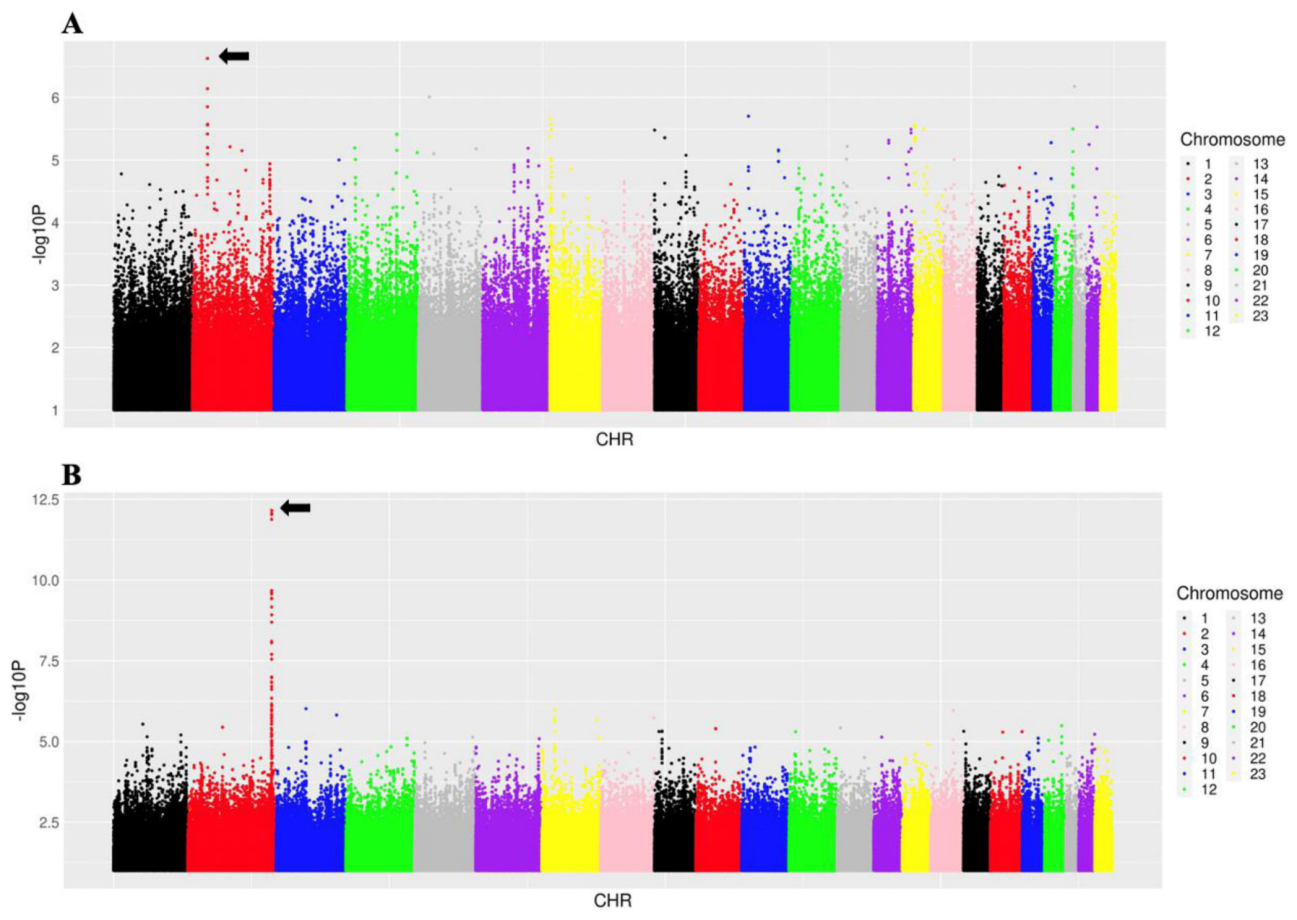
Manhattan plots of genome-wide associations with dolutegravir pharmacokinetic parameter and log_10_ bilirubin concentrations. The figure shows -log_10_ P-values for association among 284 individuals who were evaluable for genetic associations. The black arrows indicate the lowest P-value in each figure. Note the different Y-axis scales. *Panel A:* Associations with dolutegravir AUC_VAR_. The lowest P-value was *CAMKMT* rs343942 (P = 2.4 x 10^-7^). *Panel B:* Associations with log_10_ bilirubin concentrations. The lowest P-value was rs6742078 in the *UGT1A* locus (P = 7.0 x 10^-13^).

In analyses that explored genome-wide associations with AUC_VAR_, none withstood correction for multiple testing. The lowest P-value was *CAMKMT* rs343942 (β = 0.29, P = 2.4 x 10^-7^), (Figure 3A). By comparison, the lowest P-value with log_10_ bilirubin concentrations was rs6742078 in the *UGT1A* locus (β = 0.11, P = 7.0 x 10^-13^) (Figure 3B). The 10 lowest P-values for AUC_VAR_ and for log_10_ bilirubin concentrations are presented in Table 2. Results for CL_BSV_ were consistent with results for AUC_VAR_ (data not shown). For CL_BSV,_ the lowest P-value was *LOC105377607* rs201393786 on chromosome 4 (β = 0.10, P = 2.6 x 10^-7^) (Supplementary Data). As we did for *UGT1A* locus polymorphisms, we characterized associations genome-wide after adjusting for rs887829. In these analyses, the lowest P-value for dolutegravir AUC_VAR_ was *ADGRE4P* rs7256367 (β = 0.20, P = 2.6 x 10^-7^), which was also the lowest P-value for CL_BSV_ (β = -0.11, P = 2.3 x 10^-7^), and did not withstand correction for multiple testing. The 10 lowest P-values for AUC_VAR_ after adjusting for rs887829 are in Supplementary Data.

We next considered 89,716 polymorphisms previously associated with any trait in the GWAS Catalog. In these analyses, the lowest P-value for AUC_VAR_ was *CAMKMT* rs343968 (β = 0.33, P = 2.8 x 10^-6^). The 10 lowest P-values for association with AUC_VAR_ are presented in Table 3. PharmGKB and GWAS Catalog polymorphisms included in our analyses are provided in Supplementary Data. Results for CL_BSV_ were consistent with results for AUC_VAR_ (data not shown), with the lowest the lowest P-value being rs4148325 in the *UGT1A* locus (β = -0.09, P = 5.0 x 10^-6^), which is in strong LD with rs887829 (*r*^2^ = 0.99).

### Including genotype in the population pharmacokinetic model

**Figure 4:**
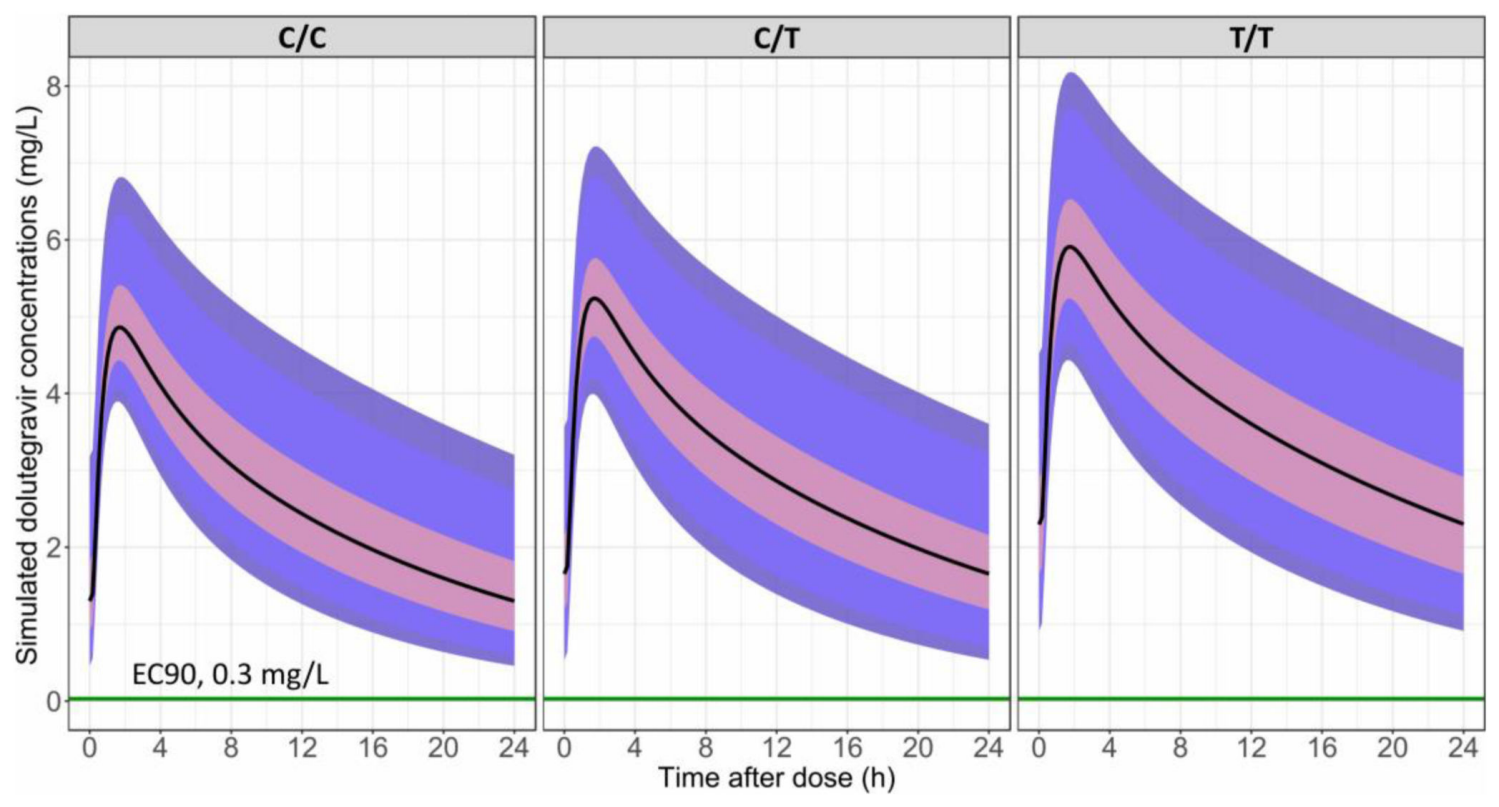
Simulated dolutegravir concentration-time profiles. Simulations are made with the final dolutegravir model and based on 1200 typical individuals categorized either as C/C, C/T or T/T for rs887829 (dolutegravir is administered at 50 mg once daily). For each panel, the solid black line in the middle represents the median simulated concentration, the pink shaded area represents the 75^th^ percentile, the blue shaded area is the 95^th^ percentile and the darkest blue at the extremes is the 97.5^th^ percentile of the simulated concentrations. The green horizontal line is dolutegravir’s effective concentration 90 (0.3 mg/L).

Among the 41 intensively sampled individuals, 26 (8 with C/C, 10 with C/T, 8 with C/T) had *UGT1A1* rs887829 genotype information and when this was tested on dolutegravir clearance, there was a graded trend towards reduced clearance for C/T and T/T individuals of borderline significance. However, when we fitted a mixture model to all available pharmacokinetic data (intensive and sparse samples) there was a significant and graded effect of *UGT1A1* rs887829 genotype on dolutegravir clearance (ΔOFV= -24.7, P < 0.00001 compared to no genotype). We estimated a clearance of 0.78 L/h for C/C, with a 10.8% and 25.9% decrease in clearance for C/T, and T/T, respectively. This included 472 individuals (188 not genotyped) who provided 742 concentrations. Simulations performed using the final model and highlighted in Figure 4 show that dolutegravir trough concentrations are highest with T/T genotypes. A VPC and a schematic of the model are in Supplementary Data.

Testing the effect of rs6736508 on dolutegravir clearance within a model that already included rs887829 only improved the model by -5.73 points (P = 0.057) and therefore, rs6736508 was not retained in the model. On the other hand, testing the effect of rs28899168 within a model that already included rs887829 improved the model by -7.42 points (P = 0.0244). However, this was not significant at the backward elimination step where we used a threshold of P < 0.01 and therefore, rs28899168 was not retained in the final model.

## Discussion

We characterised genetic associations with between-individual variability in dolutegravir exposure among participants in the ADVANCE study in South Africa. Among polymorphisms previously associated with plasma dolutegravir exposure (20–23), the UGT1A1 rs887829 T allele was associated with greater AUC_VAR_ (P = 1.8 x 10^-4^). This allele is known to be in strong linkage with the Gilbert trait decreased expression allele, UGT1A1*28, a promoter TA_n_ dinucleotide (19). Our finding supports previously reports associating UGT1A1*28 with dolutegravir concentrations (20,21,23). In prior genome-wide association studies of bilirubin concentrations, rs887829 was most strongly and consistently associated (17,24). When included in the population pharmacokinetic model, rs887829 C/T and T/T genotypes were associated with 10.8% and 25.9% decreases in dolutegravir clearance, respectively, and thus higher exposures compared to C/C individuals.

To identify novel *UGT1A* locus associations with dolutegravir exposure we used screening bilirubin concentration as a biomarker for *UGT1A1* activity. After controlling for rs887829, a second *UGT1A* locus polymorphism, rs28899168 (intronic in *UGT1A8, UGT1A9*, and *UGT1A10*) was independently associated with log_10_ bilirubin concentrations (P = 7.7 x 10^-8^, below the Bonferroni P-value cut-off of 5.9 x 10^-5^), suggesting that rs28899168 was associated with increased *UGT1A1* expression or activity independent of rs887829 (albeit with a P-value 5-log greater than that for rs887829). After adjusting for rs887829, rs28899168 was also independently associated with lower dolutegravir AUC_VAR_ (P = 0.02). Although the P-value for rs28899168 did not withstand correction for multiple comparisons, it is likely true given its independent association with bilirubin. This polymorphism has not been previously associated with dolutegravir pharmacokinetics or bilirubin.

In genome-wide analyses for associations with AUC_VAR_, the lowest P-value was *CAMKMT* rs343942 (P = 2.4 x 10^-7^), which has not been previously associated with dolutegravir pharmacokinetics. *CAMKMT* catalyses Lys-116 trimethylation in calmodulin (25), and other *CAMKMT* polymorphisms have been associated with anxiety risk (25,26). The second lowest P-value was *MIR99AHG* rs9980715 (P = 6.6 x 10^-7^). *MIR99AHG* is a potential noncoding tumor suppressor gene in lung adenocarcinoma (27). These seem unlikely to represent true associations with dolutegravir pharmacokinetics.

Previous reports have associated UGT1A1*28 rs8175347 with dolutegravir pharmacokinetics. Using data from nine Phase I and II studies involving 89 participants of European-American or African-American descent, UGT1A1*28 was associated with a 32% decrease in dolutegravir oral clearance, a 46% increase in AUC, and a 32% increase in C_max_ (20). Among 107 Japanese patients, UGT1A1*28 and UGT1A1*6 (rs4148323) were associated with increased dolutegravir concentrations (21). UGT1A1*6 is rare among Africans and Europeans but frequent among Asians. Using data from three Phase I and one Phase III clinical trials involving 93 Caucasian and Black African or Caribbean participants, UGT1A1*28 was associated with 28% increased dolutegravir plasma AUC_0–24h_ (23). The *UGT1A1* rs887829 association in our study supports previous reports. Beyond *UGT1A1, ABCG2* rs2231142 and *NR1I2* rs2472677 in combination were suggested to be associated with increased dolutegravir C_max_, while no further associations were found in *ABCG2* rs2231137, CYP3A4*22 rs35599367, CYP3A5*3 rs776746, and *NR1I2* rs1523130 (23). A Japanese study of 42 patients also found higher dolutegravir concentrations associated with *ABCG2* rs2231142 and no associations with polymorphisms with *ABCB1* rs1128503, rs1045642, rs2032582, or rs3842 (22). We found no associations with these *ABCB1, ABCG2, CYP3A4, CYP3A5* and *NR1I2* polymorphisms.

During HIV-1 treatment, plasma dolutegravir concentrations considerably exceed what is required to inhibit wild-type virus replication. Therefore, loss-of-function polymorphisms in *UGT1A1* are unlikely to increase antiviral efficacy. However, lower plasma dolutegravir exposure with the *UGT1A1* rs887829 C allele may be important in some situations, such as in patients receiving concomitant medications that increase dolutegravir clearance or decrease absorption, or in patients harbouring HIV-1 with reduced susceptibility to dolutegravir. Conversely, higher plasma dolutegravir concentrations associated with *UGT1A1* rs887829 T alleles may increase the risk of intolerability, supported by a Japanese study of 107 patients which reported a greater incidence of selected grade 1 or 2 neuropsychiatric adverse events among individuals carrying UGT1A1*6 or UGT1A1*28 (P = 0.05).

Our study had limitations. Although sample size was modest, this was the largest pharmacogenetic study of dolutegravir to date. A larger sample size may have identified novel genome-wide significant associations. We could not replicate some polymorphisms that were very infrequent in Africans. We did not directly genotype UGT1A1*28. However, we selected UGT1A1 rs887829 which is in strong linkage disequilibrium with UGT1A1*28.

In summary, two *UGT1A* locus polymorphisms were independently associated with dolutegravir AUC_VAR_ in a Black African population, one of which was expected (rs887829) and one of which was novel (rs28899168). The latter association should be replicated in other large cohorts. This study extends our understanding of dolutegravir pharmacogenetics in Africa, which is important given the widespread prescribing of dolutegravir in Africa.

## Supplementary Material

Supplementary file

## Figures and Tables

**Table 1:** Baseline characteristics of ADVANCE participants included in genetic association analyses

Characteristic	Dolutegravir recipients (n = 284)
Age in years, median (IQR)^a^	33 (27, 3 8)
Sex	
Male, n (%)	108 (38)
Female, n (%)	176 (62)
BMI, kg/m^2^, median (IQR)	23.2 (20.4, 26.9)
CD4 T-cell count in cells/mm^3^, median (IQR)	292 (163, 459)
Plasma HIV-1 RNA in copies/mL, median (IQR)	26 003 (6 044, 74 037)

a Abbreviations: IQR: Interquartile range

**Table 2:** Genetics associations with AUC_VAR_ and log_10_ bilirubin concentrations

Association analyses	Polymorphism	Gene	MAF	Beta	P value^b^
**Associations with SNPs selected *a priori* with AUC_VAR_**	rs887829^c^	*UGT1A*	0.41	0.14	1.8 x 10^-4^
	rs2472677	*NR1I2*	0.36	0.05	0.2
	rs776746	*CYP3A5*	0.19	0.04	0.44
	rs3842	*ABCB1*	0.28	-0.02	0.59
	rs1045642	*ABCB1*	0.12	-0.03	0.66
	rs1128503	*ABCB1*	0.08	0.02	0.8
	rs1523130	*NR1I2*	0.06	-0.01	0.9
	rs2231137	*ABCG2*	0.06	-0.01	0.94
**Genome-wide associations**	**AUC_VAR_**				
	rs343942	*CAMKMT*	0.12	0.29	2.4 x 10^-7^
	rs9980715	*MIR99AHG*	0.29	0.19	6.6 x 10^-7^
	rs75466245	*CAMKMT*	0.05	0.38	7.2 x 10^-7^
	rs1038692137	Intergenic	0.19	0.23	9.8 x 10^-7^
	rs343960	*CAMKMT*	0.05	0.38	1.4 x 10^-6^
	rs76142931	Intergenic	0.06	-0.37	2.0 x 10^-6^
	rs140435425^c^	Intergenic	0.07	-0.34	2.2 x 10^-6^
	rs112238172	Intergenic	0.11	0.27	2.7 x 10^-6^
	rs111066265	*MAD1L1*	0.21	-0.21	2.7 x 10^-6^
	rs343950	*CAMKMT*	0.06	0.34	2.7 x 10^-6^
	**Log_10_ bilirubin**				
	rs6742078	*UGT1A*	0.4	0.11	7.0 x 10^-13^
	rs887829^c^	*UGT1A*	0.41	0.12	8.6 x 10^-13^
	rs4148325	*UGT1A*	0.41	0.12	9.4 x 10^-13^
	rs4148324	*UGT1A*	0.41	0.11	1.3 x 10^-12^
	rs11888459^c^	*UGT1A*	0.38	0.1	2.1 x 10^-10^
	rs28899168	*UGT1A*	0.15	-0.14	2.5 x 10^-10^
	rs34352510	*UGT1A*	0.37	0.1	2.7 x 10^-10^
	rs7604115	*UGT1A*	0.37	0.1	3.7 x 10^-10^
	rs11673726^c^	*UGT1A*	0.37	0.1	3.8 x 10^-10^
	rs7564935	*UGT1A*	0.36	0.1	6.8 x 10^-10^

a Abbreviations: AUC_VAR_, unexplained variability in population estimates of AUC values; MAF, Minor allele frequency.

b Significance threshold was 5.6 x 10^-3^ for the subset of nine polymorphisms selected *a priori*. Genome-wide significance threshold was 5.0 x 10^-8^. For each pharmacokinetic parameter, the 10 lowest P-values are shown.

c These polymorphisms were in linkage with other polymorphisms in our imputed data, which gave identical association results. These included: rs140435425 with rs139714988, rs144113286 and rs150029007; rs887829 with rs1976391 and rs1368812138; rs11888459 with rs10178992; and rs11673726 with rs3771341.

**Table 3:** Associations between unexplained variability in population estimates of AUC values (AUC_VAR_) and polymorphisms previously associated with any trait in the GWAS Catalog

Polymorphism	Gene	Chromosome	MAF^a^	Beta	P value^b^	GWAS Catalog trait
rs343968	*CAMKMT*	2	0.07	0.33	2.8 x 10^-6^	Height
rs4418728	Intergenic	10	0.20	-0.19	8.4 x 10^-5^	Triglyceride levels
rs4148325	*UGT1A5*	2	0.41	0.15	1.2 x 10^-4^	Bilirubin concentrations
rs4148324	*UGT1A10*	2	0.41	0.14	1.3 x 10^-4^	Bilirubin concentrations
rs10473629	Intergenic	5	0.13	0.22	1.3 x 10^-4^	Self-reported math ability
rs35207189	Intergenic	13	0.37	-0.15	1.6 x 10^^-4^^	Household income (MTAG)
rs887829^c^	UGT1A1	2	0.41	0.14	1.8 x 10^^-4^^	Bilirubin concentrations

a Abbreviations: MAF, Minor allele frequency.

b The seven lowest P values for associations with GWAS Catalog traits are shown.

c Polymorphism rs887829 was in complete linkage with rs1976391 in our imputed genotype data, so gave identical results.
