# Supplementary material for: Pharmacogenetics of dolutegravir plasma exposure among Southern Africans living with HIV
Source: J Infect Dis. Author manuscript; Available in PMC 2022 Nov 7. (PMC9624457; doi:10.1093/infdis/jiac174)
Supplement: Supplementary file [file EMS145648-supplement-Supplementary_file.pdf]

Additional methodology

Detection of implausible sparse values during population pharmacokinetic analysis;

For each participant who was sparsely sampled, implausible concentrations were identified and withdrawn from the analysis based on the absolute value of conditional weighted residual (CWRES) being greater than 4. CWRES follows a normal distribution with mean 0 and variance 1. Hence, for a model that fits adequately, we expect less than 0.01% of data to have |CWRES| >4. A total of 23 sparsely sampled participants were excluded.

Additional figures

Figure S1

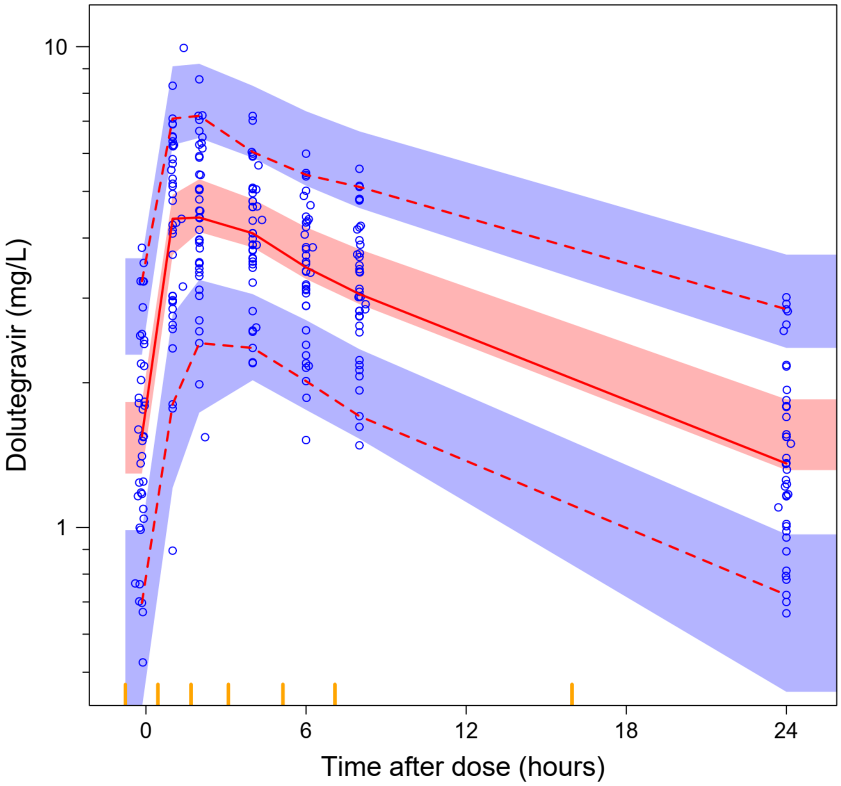

Visual predictive check of the final model fitted to intense samples only (without genetic information). Blue circles represent observed plasma concentrations. The solid line in the middle represents the median observed concentration, the dashed lines below and above represent the 5th and 95th percentiles of the observed concentrations, respectively. The shaded areas around each line represent the 95% confidence interval for the same percentiles based on simulations with the model.

Figure S2

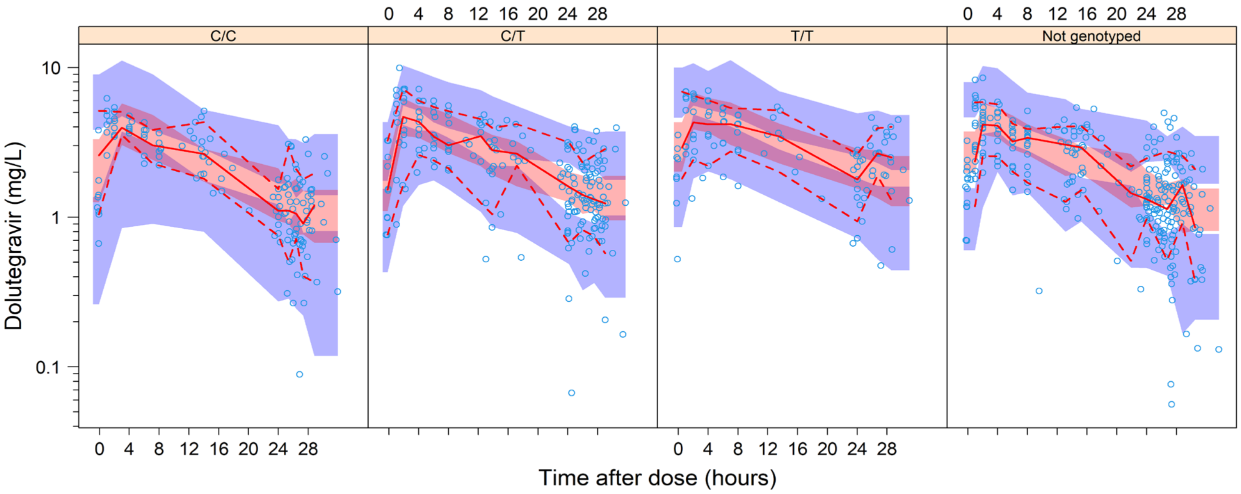

Visual predictive check of the mixture model including *UGT1A1* rs887829 genotype effect on clearance. Blue circles represent observed plasma concentrations. The solid line in the middle represents the median observed concentration, the dashed lines below and above represent the 5th and 95th percentiles of the observed concentrations, respectively. The shaded areas around each line represent the 95% confidence interval for the same percentiles based on simulations with the model.

Figure S3

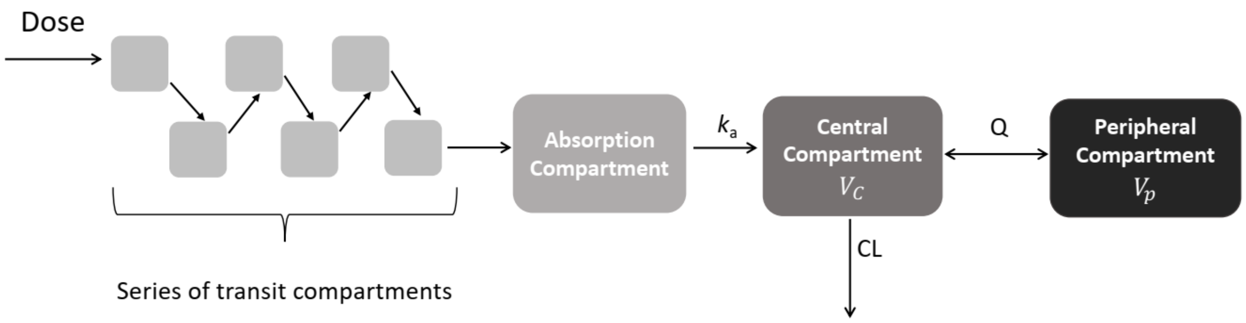

Schematic of the dolutegravir structural model. Once administered, the dose of dolutegravir goes through a series of transit compartments (characterized by a mean transit time and number of compartments) before being absorbed into the central compartment. It then distributes to a peripheral compartment and is eliminated from the central compartment with first-order kinetics. k_a, absorption rate constant; V_c, central volume of distribution; V_p, peripheral volume of distribution; Q, intercompartmental clearance; CL, central clearance.

Figure S4

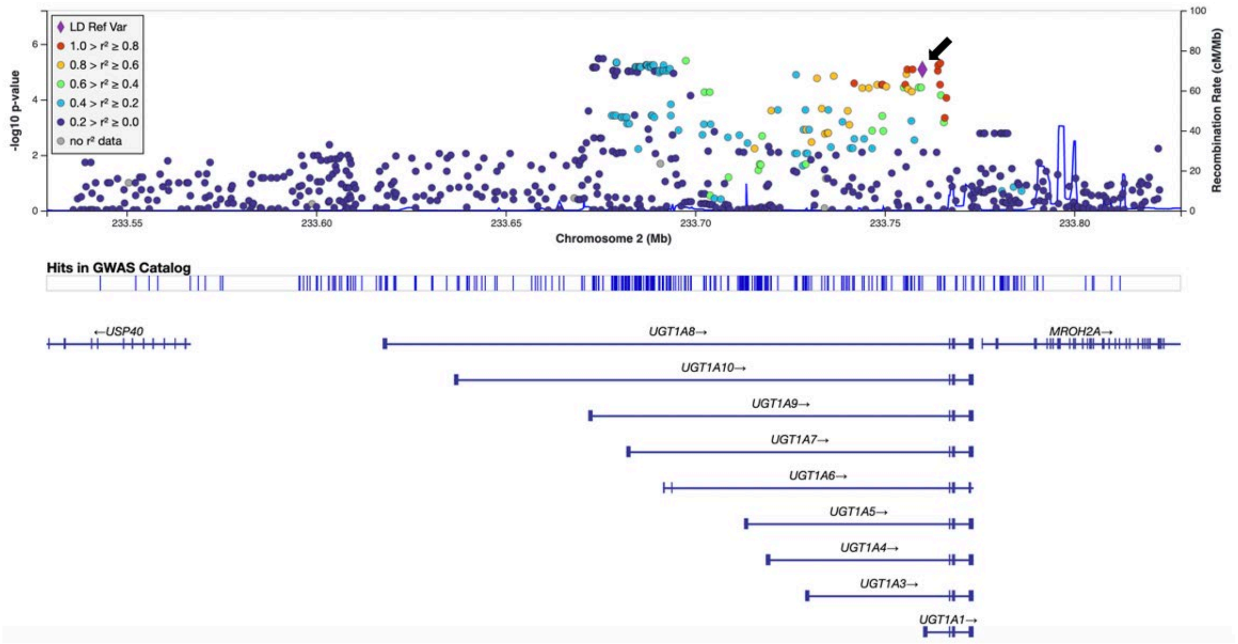

LocusZoom plot of UGT1A locus associations with between-individual variability in dolutegravir clearance. The figure shows -log10 P-values for associations of 853 polymorphisms in the *UGT1A* locus (±50 kB in either direction) among 284 individuals who were evaluable for genetic associations. The purple diamond identifies *UGT1A1* rs887829 which we selected to be the reference polymorphism for linkage disequilibrium (LD) values because it has been most consistently associated with bilirubin concentrations in prior genome-wide association studies. The lowest P-value was rs201393786 in *LOC105377607* (P = 2.6 x 10^{-7}).

Figure S5

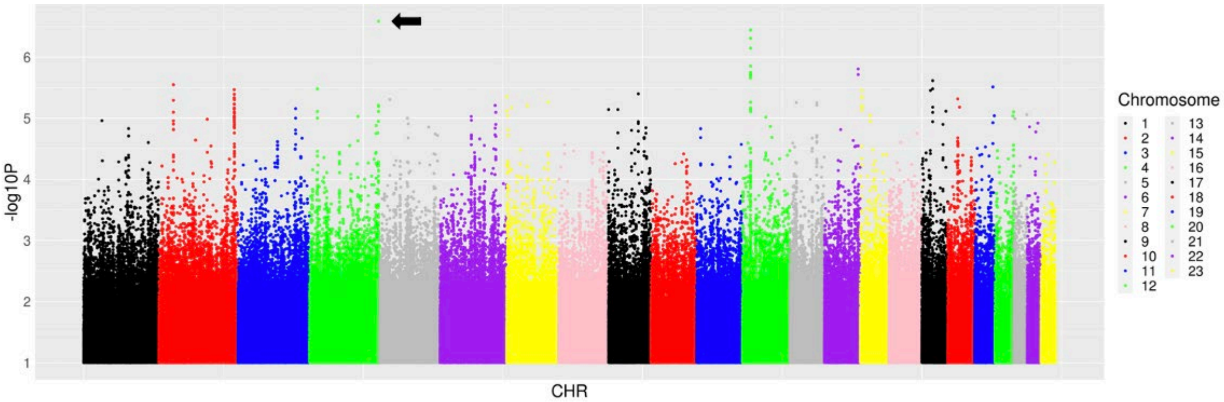

Manhattan plot of genome-wide associations with between-individual variability in dolutegravir clearance. The figure shows -$\log_{10}$ P-values for association among 284 individuals who were evaluable for genetic associations. The arrow indicate the lowest P-value. The lowest P-value was rs201393786 in *LOC105377607* (P = 2.6 x 10^{-7}).

Additional tables

Table S1: Final population parameter estimates for dolutegravir

Parameter description	Typical Value (95% CI)[a]	
	**Intensive samples only *n*=41 participants**	**Intensive and sparse *n*=472 participants*** (With genetic information included)
Clearance (CL) (L/h)[c]	0.732 (0.666–0.801)	0.786 (0.730–0.846)
Effect of Homozygous for *UGT1A1* rs887829 on CL (%)		-25.9% (-33.8– -16.7)
Effect of Heterozygous for *UGT1A1* rs887829 on CL (%)		-10.8% (-18.4– -2.09)
Central volume (L) [c]	12.2 (1.12–13.4)	10.5 (8.44–12.3)
Inter-compartmental clearance (L/h) [c]	0.509 (0.252–3.86)	1.29 (0.524–2.81)
Peripheral volume (L) [c]	5.87 (1.74–41.6)	3.77 (2.40–5.36)
Relative bioavailability (*F*)	1 Fixed	1 Fixed
Absorption mean transit time (MTT) (h)	0.166 (0.00625–0.396)	0.180 (0.086–0.292)
Number of Transit compartments (n)	7.85 (2.64–22.3)	5 Fixed
Absorption rate constant (ka) (/h)	2.41 (0.242–4.28)	1.67 (1.17–2.30)
**Parameter Variability (% CV) [b]**		
Between-subject variability in CL	22.3% (14.1–27.8)	27.9% (24.6–31.6)
Between-occasion variability in F	31.6 % (21.4–41.7)	38.7% (31.9–45.1)
Between-occasion variability in MTT	130 % (63.5–445)	184% (130–251)
Between-occasion variability in ka	69.9 % (30.9–97.1)	60.7% (43.8–78.6)
**Residual unexplained variability**		
Proportional error (%)	7.41 (4.51–10.2)	7.52 (5.89–9.35)
Additive error (mg/L)	0.191 (0.0602–0.256)	0.180 (0.127–0.232)

[a] 95% confidence intervals were obtained by sampling importance resampling

FFM, fat-free mass (calculated according to Janmahasatian et al);(1) %CV, coefficient of variation.

[b] Calculated by $\%CV = \sqrt{\omega}.100\%$.

[c] Clearance and volume of distribution parameters were scaled with fat-free mass (FFM) using the exponents 0.75 and 1, respectively. The typical values reported here refer to an individual with an FFM of 47 kg.

*Of these, 384 were genotyped and those with missing genotype were assigned one via mixture modeling as described here (2)

Table S2: Associations between unexplained variability (between-individual variability) in the population parameter estimates of dolutegravir clearance (CL_{BSV}) and selected polymorphisms of potential relevance to dolutegravir

Polymorphism	Gene	MAF	Beta	P value[b]
rs887829[c]	*UGT1A*	0.41	-0.09	8.4 x 10^{-6}
rs2472677	*NR1I2*	0.36	-0.02	0.28
rs3842	*ABCB1*	0.28	0.01	0.64
rs776746	*CYP3A5*	0.19	-0.01	0.67
rs1128503	*ABCB1*	0.08	-0.01	0.75
rs1523130	*NR1I2*	0.06	4.2 x 10^{-3}	0.92
rs2231137	*ABCG2*	0.06	3.4 x 10^{-3}	0.94
rs1045642	*ABCB1*	0.12	1.6 x 10^{-3}	0.96

[a] Abbreviations: MAF, Minor allele frequency.

[b] Significance threshold was 5.6 x 10^{-3} for the subset of nine polymorphisms.

[c] Polymorphism rs887829 was in complete linkage with rs1976391 in our imputed genotype data, so gave identical results.

Table S3: Associations between unexplained variability (between-individual variability) in the population parameter estimates of dolutegravir clearance (CL_{BSV}) and polymorphisms previously associated with any trait in the GWAS Catalog

Polymorphism	Gene	Chromosome	MAF[a]	Beta	P value[b]	GWAS Catalog trait
rs4148325	*UGT1A3*	2	0.41	-0.09	5.0 x10^{-6}	Bilirubin concentrations
rs4148324	*UGT1A3*	2	0.41	-0.09	5.9 x10^{-6}	Bilirubin concentrations
rs887829[c]	*UGT1A3*	2	0.41	-0.09	8.4 x10^{-6}	Bilirubin concentrations
rs6742078	*UGT1A3*	2	0.4	-0.09	9.4 x10^{-6}	Bilirubin concentrations
rs343968	*CAMKMT*	2	0.07	-0.16	1.1 x 10^{-5}	Height
rs6747843	*UGT1A3*	2	0.27	-0.09	1.3 x 10^{-5}	Bilirubin concentrations
rs7564935	*UGT1A3*	2	0.36	-0.09	1.5 x 10^{-5}	Bilirubin concentrations

[a] Abbreviations: MAF, Minor allele frequency.

[b] The seven lowest P values for associations with GWAS Catalog traits are shown.

[c] Polymorphism rs887829 was in complete linkage with rs1976391 in our imputed genotype data, so gave identical results.

Table S4: Genome-wide associations with unexplained variability (between-individual variability) in the population parameter estimates of dolutegravir clearance (CL_{BSV})

Polymorphism	Gene	MAF[a]	Beta	P value[b]
rs201393786	*LOC105377607*	0.49	0.1	2.6 x 10^{-7}
rs7298064	*AEBP2*	0.21	0.12	3.6 x 10^{-7}
rs10841238[c]	*AEBP2*	0.21	0.12	5.0 x 10^{-7}
rs10770496	*AEBP2*	0.19	0.12	7.2 x 10^{-7}
rs7958449	*AEBP2*	0.16	0.12	1.4 x 10^{-6}
rs4900552	Intergenic	0.11	0.16	1.6 x 10^{-6}
rs10841224	*AEBP2*	0.19	0.12	1.8 x 10^{-6}
rs7971277	*AEBP2*	0.18	0.12	1.9 x 10^{-6}
rs4906258	Intergenic	0.1	0.17	1.9 x 10^{-6}

[a] Abbreviations: MAF, Minor allele frequency.

[b] Genome-wide significance threshold was 5.0 x 10^{-8}. The 10 lowest P-values are shown.

[c] These polymorphisms were in linkage with other polymorphisms in our imputed data, which gave identical association results, rs10841238 with rs7298006.

Table S5: GWAS with dolutegravir pharmacokinetic parameters adjusted for *UGT1A1* rs887829

Polymorphism	Gene	MAF	Beta	P value
**AUC$_{VAR}$**				
rs7256367	*ADGRE4P*	0.29	0.20	2.6×10^{-7}
rs583310	Intergenic	0.14	-0.25	4.9×10^{-7}
rs1352224	*LINC02462*	0.29	0.19	5.8×10^{-7}
rs1486986	*LINC02462*	0.29	0.19	5.8×10^{-7}
rs1578706247	Intergenic	0.29	0.19	5.8×10^{-7}
**CL$_{BSV}$**				
rs7256367	*ADGRE4P*	0.29	-0.11	2.3×10^{-7}
rs201393786	*LOC105377607*	0.49	0.09	6.2×10^{-7}
rs1352223	*LINC02462*	0.29	-0.10	9.3×10^{-7}
rs1256609206	*RAB28*	0.29	-0.10	9.3×10^{-7}
rs1486986	*LINC02462*	0.29	-0.10	9.3×10^{-7}
**Log$_{10}$ bilirubin**				
rs28899168	*UGT1A*	0.15	-0.11	7.7×10^{-8}
rs374147899	*COL11A1*	0.31	0.08	6.3×10^{-7}
rs114707025	*GPAT3*	0.06	-0.16	6.5×10^{-7}
rs877736	*PPP2R2B*	0.08	0.14	8.7×10^{-7}
rs9853252	Intergenic	0.05	0.16	9.2×10^{-7}

[a] Abbreviations: AUC$_{VAR}$, unexplained variability in population estimates of AUC values; CL$_{BSV}$, unexplained variability (between-individual variability) in the population parameter estimates of dolutegravir clearance; MAF, Minor allele frequency.

[b] Genome-wide significance threshold was $P < 5.0 \times 10^{-8}$.

For each pharmacokinetic parameter, the top five lowest P-values are shown.

Table S6: Polymorphisms previously associated with any trait from the GWAS Catalog included in our analyses

Polymorphism	Gene	Chromosome	MAF	Beta	P value	Disease trait
**AUCVAR**						
rs343968	*CAMKMT*	2	0.07	0.33	2.8×10^{-6}	Height
rs4418728	Intergenic	10	0.20	-0.19	8.4×10^{-5}	Triglyceride levels
rs4148325	*UGT1A5*	2	0.41	0.15	1.2×10^{-4}	Bilirubin concentrations
rs4148324	*UGT1A10*	2	0.41	0.14	1.3×10^{-4}	Bilirubin concentrations
rs10473629	Intergenic	5	0.13	0.22	1.3×10^{-4}	Self-reported math ability
rs35207189	Intergenic	13	0.37	-0.15	1.6×10^{-4}	Household income (MTAG)
rs887829	*UGT1A1*	2	0.41	0.14	1.8×10^{-4}	Bilirubin concentrations
rs111741722	*UGT1A5*	2	0.41	0.14	1.8×10^{-4}	Blood protein levels
rs2069568	*SLA*	8	0.19	-0.17	1.8×10^{-4}	Eosinophil counts
rs10929301	*UGT1A5*	2	0.30	-0.16	1.8×10^{-4}	Bilirubin concentrations
rs3755319	*UGT1A8*	2	0.30	-0.16	1.8×10^{-4}	Bilirubin concentrations
rs2516599	*NID2*	14	0.48	0.13	1.8×10^{-4}	Lung function (FEV1/FVC)
rs9376091	Intergenic	6	0.20	0.18	2.2×10^{-4}	Neutrophil count
rs6742078	*UGT1A8*	2	0.40	0.14	2.5×10^{-4}	Bilirubin concentrations
rs9387925	Intergenic	6	0.35	-0.14	2.5×10^{-4}	Intelligence (MTAG)
rs7821092	*RP1L1*	8	0.44	-0.14	2.5×10^{-4}	LDL cholesterol
rs6747843	*UGT1A10*	2	0.27	0.15	2.6×10^{-4}	Bilirubin concentrations
rs10804990	*TBC1D14*	4	0.29	0.15	2.7×10^{-4}	Mean corpuscular volume
rs1122794	*FAM234A*	16	0.20	0.16	2.8×10^{-4}	Mean corpuscular hemoglobin
rs1006548	*FANCA*	16	0.28	-0.15	2.8×10^{-4}	Low tan response
rs7564935	*UGT1A6*	2	0.36	0.14	2.9×10^{-4}	Bilirubin concentrations
rs11695484	*UGT1A8*	2	0.31	0.14	3.0×10^{-4}	Bilirubin concentrations
rs4332347	*DISC1*	1	0.20	-0.16	3.0×10^{-4}	Adolescent idiopathic scoliosis
rs17864701	*UGT1A10*	2	0.30	0.14	3.2×10^{-4}	Bilirubin concentrations
rs7102705	Intergenic	11	0.33	0.15	3.3×10^{-4}	Breast size
rs10866054	*CADPS*	3	0.41	-0.14	3.5×10^{-4}	Risk-taking tendency (4-domain principal component model)
rs2885296	*UGT1A7*	2	0.30	0.14	3.6×10^{-4}	Bilirubin concentrations
rs5510	*SERPINA4*	14	0.42	-0.13	3.8×10^{-4}	Protein levels in obesity
rs6714634	*UGT1A10*	2	0.30	0.14	4.2×10^{-4}	Bilirubin concentrations
rs10859277	*LINC01619*	12	0.24	0.16	4.3×10^{-4}	Eosinophil counts
rs2268796	*SRD5A2*	2	0.29	0.14	4.3×10^{-4}	Blood protein levels
**AUCVAR**						

Polymorphism	Gene	Chromosome	MAF	Beta	P value	Disease trait
rs12988520	*UGT1A8*	2	0.26	0.15	4.5×10^{-4}	Bilirubin concentrations
rs10929302	*UGT1A8*	2	0.29	0.14	4.6×10^{-4}	Bilirubin concentrations
rs2068888	Intergenic	10	0.21	-0.16	4.6×10^{-4}	Sex hormone-binding globulin levels adjusted for BMI
rs11597947	*ZMIZ1*	10	0.08	-0.23	4.7×10^{-4}	Monocyte count
rs34352510	*UGT1A10*	2	0.37	0.13	4.7×10^{-4}	Bilirubin concentrations
rs11080055	*TMEM97*	17	0.49	-0.13	4.8×10^{-4}	Blood protein levels
rs10819335	Intergenic	9	0.47	0.13	4.9×10^{-4}	Monocyte count
rs168643	Intergenic	5	0.15	0.19	4.9×10^{-4}	Diastolic blood pressure
rs12611922	*BAZ2B*	2	0.39	0.14	5.0×10^{-4}	Neutrophil percentage of white cells
rs11638908	*MEGF11*	15	0.05	0.26	5.1×10^{-4}	Mean corpuscular hemoglobin
rs3008892	*WDR78*	1	0.08	0.25	5.2×10^{-4}	Height
rs1595217	Intergenic	13	0.32	-0.14	5.3×10^{-4}	Reaction time
rs10178992	*UGT1A5*	2	0.38	0.13	5.4×10^{-4}	Bilirubin concentrations
rs11888459	*UGT1A7*	2	0.38	0.13	5.4×10^{-4}	Bilirubin concentrations
rs10178992	*UGT1A10*	2	0.38	0.13	5.4×10^{-4}	Bilirubin concentrations
rs11888459	*UGT1A2P*	2	0.38	0.13	5.4×10^{-4}	Bilirubin concentrations
rs10178992	*UGT1A8*	2	0.38	0.13	5.4×10^{-4}	Bilirubin concentrations
rs11888459	*UGT1A5*	2	0.38	0.13	5.4×10^{-4}	Bilirubin concentrations
rs10937763	*TBC1D14*	4	0.30	0.14	5.7×10^{-4}	Mean platelet volume
**CL_{BSV}**						
rs4148325	*UGT1A3*	2	0.41	-0.09	5.0×10^{-6}	Bilirubin concentrations
rs4148324	*UGT1A3*	2	0.41	-0.09	5.9×10^{-6}	Bilirubin concentrations
rs887829	*UGT1A3*	2	0.41	-0.09	8.4×10^{-6}	Bilirubin concentrations
rs111741722	*UGT1A3*	2	0.41	-0.09	8.4×10^{-6}	Blood protein levels
rs6742078	*UGT1A3*	2	0.40	-0.09	9.4×10^{-6}	Bilirubin concentrations
rs343968	*CAMKMT*	2	0.07	-0.16	1.1×10^{-5}	Height
rs6747843	*UGT1A3*	2	0.27	-0.09	1.3×10^{-5}	Bilirubin concentrations
rs7564935	*UGT1A3*	2	0.36	-0.09	1.5×10^{-5}	Bilirubin concentrations
rs34352510	*UGT1A3*	2	0.37	-0.08	2.6×10^{-5}	Bilirubin concentrations
rs11673726	*UGT1A3*	2	0.37	-0.08	3.0×10^{-5}	Bilirubin concentrations
rs3771341	*UGT1A3*	2	0.37	-0.08	3.0×10^{-5}	Bilirubin concentrations
rs7604115	*UGT1A3*	2	0.37	-0.08	3.0×10^{-5}	Bilirubin concentrations
rs11888459	*UGT1A3*	2	0.38	-0.08	3.0×10^{-5}	Bilirubin concentrations
rs10178992	*UGT1A3*	2	0.38	-0.08	3.0×10^{-5}	Bilirubin concentrations
**CL_{BSV}**						

Polymorphism	Gene	Chromosome	MAF	Beta	P value	Disease trait
rs2885296	*UGT1A3*	2	0.30	-0.08	3.5×10^{-5}	Bilirubin concentrations
rs3755319	*UGT1A3*	2	0.30	0.09	3.7×10^{-5}	Bilirubin concentrations
rs10929301	*UGT1A3*	2	0.30	0.09	3.7×10^{-5}	Bilirubin concentrations
rs11695484	*UGT1A3*	2	0.31	-0.08	3.9×10^{-5}	Bilirubin concentrations
rs17864701	*UGT1A3*	2	0.30	-0.08	3.9×10^{-5}	Bilirubin concentrations
rs6714634	*UGT1A3*	2	0.30	-0.08	4.2×10^{-5}	Bilirubin concentrations
rs61912333	Intergenic	12	0.20	0.09	5.1×10^{-5}	Diastolic blood pressure
rs10929302	*UGT1A3*	2	0.29	-0.08	5.3×10^{-5}	Bilirubin concentrations
rs17863787	*UGT1A9*	2	0.19	-0.10	5.5×10^{-5}	Bilirubin concentrations
rs12988520	*UGT1A9*	2	0.26	-0.09	7.3×10^{-5}	Bilirubin concentrations
rs73339979	Intergenic	17	0.20	0.09	1.0×10^{-4}	Total cholesterol levels
rs5510	*SERPINA4*	14	0.42	0.07	1.4×10^{-4}	Protein levels in obesity
rs2516599	*NID2*	14	0.48	-0.07	1.4×10^{-4}	Lung function (FEV1/FVC)
rs146543016	*CRTC1*	19	0.14	0.11	1.5×10^{-4}	Self-reported math ability
rs674514	*LOC105377392*	4	0.38	-0.08	1.5×10^{-4}	Neuroticism
rs708100	*TMEM199*	17	0.49	-0.07	1.7×10^{-4}	Blood protein levels
rs11638908	*MEGF11*	15	0.05	-0.15	2.1×10^{-4}	Mean corpuscular hemoglobin
rs9376091	Intergenic	6	0.20	-0.09	2.1×10^{-4}	Neutrophil count
rs10804990	*TBC1D14*	4	0.29	-0.08	2.2×10^{-4}	Mean corpuscular volume
rs704	*VTN*	17	0.50	-0.07	2.2×10^{-4}	Blood protein levels
rs10866054	*CADPS*	3	0.41	0.08	2.2×10^{-4}	Risk-taking tendency (4-domain principal component model)
rs4418728	Intergenic	10	0.20	0.09	2.3×10^{-4}	Triglyceride levels
rs203425	*EEF1DP3*	13	0.07	0.13	2.5×10^{-4}	DNA methylation variation (age effect)
rs13401281	*UGT1A4*	2	0.43	0.07	2.5×10^{-4}	Total bilirubin concentrations
rs1006548	*FANCA*	16	0.28	0.08	2.5×10^{-4}	Low tan response
rs9892407	Intergenic	17	0.21	0.09	2.6×10^{-4}	Blood protein levels
rs2069568	*TG*	8	0.19	0.09	2.9×10^{-4}	Eosinophil counts
rs241775	Intergenic	17	0.46	-0.07	2.9×10^{-4}	Blood protein levels
rs2599469	Intergenic	19	0.44	-0.07	3.0×10^{-4}	Adolescent idiopathic scoliosis
rs11080055	*TMEM97*	17	0.49	0.07	3.4×10^{-4}	Blood protein levels
rs9387925	Intergenic	6	0.35	0.07	3.7×10^{-4}	Intelligence (MTAG)
rs2070959	*UGT1A9*	2	0.16	-0.10	3.8×10^{-4}	Bilirubin concentrations
rs10173355	*UGT1A9*	2	0.16	-0.10	3.8×10^{-4}	Bilirubin concentrations
**CL_{BSV}**						
rs10819335	Intergenic	9	0.47	-0.07	3.9×10^{-4}	Monocyte count

Polymorphism	Gene	Chromosome	MAF	Beta	P value	Disease trait
rs10179091	*UGT1A3*	2	0.48	0.07	3.9×10^{-4}	Bilirubin concentrations
rs35207189	Intergenic	13	0.37	0.07	4.0×10^{-4}	Household income (MTAG)

[a] Abbreviations: AUC_{VAR}, unexplained variability in population estimates of AUC values; CL_{BSV}, unexplained variability (between-individual variability) in the population parameter estimates of dolutegravir clearance; MAF, Minor allele frequency.

Table S7: Polymorphisms included in our analyses from PharmGKB

Polymorphism	Gene	Chromosome	MAF	Beta	P value
**AUCVAR**					
rs887829	*UGT1A6*	2	0.410	0.143	1.8×10^{-4}
rs6295	Intergenic	5	0.421	-0.089	0.020
rs20455	*KIF6*	6	0.178	0.097	0.054
rs4917639	*CYP2C9*	10	0.174	0.070	0.130
rs4673993	*ATIC*	2	0.067	-0.111	0.138
rs7270101	*ITPA*	20	0.092	-0.084	0.181
rs4713916	*FKBP5*	6	0.093	0.083	0.185
rs16960228	*PRKCA*	17	0.380	-0.048	0.219
rs4961	*ADD1*	4	0.073	-0.091	0.223
rs1695	*GSTP1*	11	0.486	-0.045	0.225
rs1801133	*MTHFR*	1	0.090	0.084	0.236
rs1051730	*CHRNA3*	15	0.053	-0.091	0.267
rs924607	*LOC100996325*	5	0.072	0.080	0.300
rs3812718	*SCN1A*	2	0.287	-0.040	0.336
rs489693	Intergenic	18	0.438	0.036	0.345
rs678849	*OPRD1*	1	0.237	0.041	0.353
rs3212986	*ERCC1*	19	0.221	-0.040	0.359
rs1954787	*GRIK4*	11	0.120	0.050	0.397
rs11881222	*IFNL3*	19	0.382	0.032	0.433
rs4444903	*EGF*	4	0.227	-0.033	0.440
rs776746	*CYP3A5*	7	0.185	0.039	0.440
rs12777823	Intergenic	10	0.293	-0.034	0.447
rs12979860	*IFNL4*	19	0.414	0.028	0.460
rs8050894	*VKORC1*	16	0.243	-0.030	0.482
rs8099917	Intergenic	19	0.053	0.059	0.500
rs1800629	Intergenic	6	0.154	-0.032	0.544
rs510769	*OPRM1*	6	0.196	0.028	0.555
rs1051266	*SLC19A1*	21	0.276	-0.025	0.560
rs7294	*VKORC1*	16	0.408	0.021	0.573
rs3745274	*CYP2B6*	19	0.380	-0.021	0.588
rs6065	*GP1BA*	17	0.167	0.026	0.605
rs7297610	Intergenic	12	0.363	0.019	0.628
rs11615	*ERCC1*	19	0.056	-0.040	0.629
rs1045642	*ABCB1*	7	0.120	-0.025	0.656
**AUCVAR**					
rs2236857	*OPRD1*	1	0.220	-0.017	0.692

Polymorphism	Gene	Chromosome	MAF	Beta	P value
rs578776	*CHRNA3*	15	0.405	0.013	0.747
rs25487	*XRCC1*	19	0.103	-0.018	0.772
rs3766951	*OPRD1*	1	0.312	0.010	0.792
rs1799930	*NAT2*	8	0.235	-0.010	0.824
rs4680	*COMT*	22	0.332	-0.006	0.876
rs10306114	*PTGS1*	9	0.132	0.008	0.879
rs2740574	*LOC110366354*	7	0.252	0.006	0.886
rs2298383	*ADORA2A-AS1*	22	0.290	0.005	0.904
rs2359612	*VKORC1*	16	0.260	0.004	0.911
rs1800566	*NQO1*	16	0.176	-0.005	0.916
rs1042713	*ADRB2*	5	0.472	0.003	0.940
rs1051740	*EPHX1*	1	0.218	0.002	0.956
rs17782313	Intergenic	18	0.252	-0.002	0.957
rs11045879	*SLCO1B1*	12	0.065	-0.003	0.965
rs7412	*APOE*	19	0.168	0.002	0.974
rs2011425	*UGT1A8*	2	0.083	0.001	0.989
**CL_{BSV}**					
rs887829	*UGT1A3*	2	0.410	-0.089	8.6×10^{-6}
rs6295	Intergenic	5	0.421	0.049	0.015
rs20455	*LOC107986594*	6	0.178	-0.046	0.079
rs7270101	*ITPA*	20	0.092	0.054	0.097
rs4917639	*CYP2C9*	10	0.174	-0.037	0.124
rs8050894	*VKORC1*	16	0.243	0.031	0.168
rs1051730	*CHRNA3*	15	0.053	0.057	0.185
rs4713916	*FKBP5*	6	0.093	-0.043	0.195
rs4673993	*ATIC*	2	0.067	0.051	0.197
rs924607	*LOC105374608*	5	0.072	-0.052	0.201
rs3812718	*SCN1A*	2	0.287	0.028	0.202
rs16960228	*PRKCA*	17	0.380	0.025	0.212
rs4961	*ADD1*	4	0.073	0.044	0.260
rs1695	*GSTP1*	11	0.486	0.021	0.272
rs7294	*VKORC1*	16	0.408	-0.022	0.277
rs4444903	*EGF*	4	0.227	0.022	0.327
rs3766951	*OPRD1*	1	0.312	-0.020	0.348
rs489693	Intergenic	18	0.438	-0.018	0.359
**CL_{BSV}**					
rs678849	*OPRD1*	1	0.237	-0.021	0.371
rs11881222	*IFNL3*	19	0.382	-0.019	0.380

Polymorphism	Gene	Chromosome	MAF	Beta	P value
rs7297610	Intergenic	12	0.363	-0.017	0.396
rs1801133	*MTHFR*	1	0.090	-0.030	0.413
rs1954787	*GRIK4*	11	0.120	-0.021	0.505
rs8099917	Intergenic	19	0.053	-0.029	0.528
rs12777823	Intergenic	10	0.293	0.013	0.587
rs11615	*ERCC1*	19	0.056	0.022	0.608
rs1042713	*ADRB2*	5	0.472	0.009	0.625
rs25487	*XRCC1*	19	0.103	0.016	0.626
rs3212986	*CD3EAP*	19	0.221	0.011	0.628
rs776746	*ZSCAN25*	7	0.185	-0.011	0.666
rs4680	*COMT*	22	0.332	0.008	0.682
rs3745274	*CYP2B6*	19	0.380	0.007	0.726
rs2011425	*UGT1A9*	2	0.083	0.012	0.743
rs1800629	Intergenic	6	0.154	0.009	0.749
rs2359612	*VKORC1*	16	0.260	-0.007	0.752
rs1799930	*NAT2*	8	0.235	0.007	0.753
rs7412	*APOE*	19	0.168	0.007	0.782
rs17782313	Intergenic	18	0.252	0.006	0.783
rs6065	*GP1BA*	17	0.167	-0.007	0.784
rs12979860	*IFNL4*	19	0.414	-0.005	0.789
rs2298383	*ADORA2A-AS1*	22	0.290	-0.006	0.795
rs1800566	*NQO1*	16	0.176	-0.006	0.826
rs10306114	*PTGS1*	9	0.132	-0.004	0.885
rs1051266	*SLC19A1*	21	0.276	0.003	0.890
rs2740574	*LOC110366354*	7	0.252	0.003	0.892
rs578776	*CHRNA3*	15	0.405	-0.002	0.923
rs2236857	*OPRD1*	1	0.220	-0.002	0.932
rs510769	*OPRM1*	6	0.196	0.002	0.942
rs1051740	*EPHX1*	1	0.218	-0.001	0.956
rs1045642	*ABCB1*	7	0.120	0.002	0.957
rs11045879	*SLCO1B1*	12	0.065	0.001	0.976

[a] Abbreviations: $AUC_{VAR,}$ unexplained variability in population estimates of AUC values; CL_{BSV}, unexplained variability (between-individual variability) in the population parameter estimates of dolutegravir clearance; MAF, Minor allele frequency.

References

1. Janmahasatian S, Duffull SB, Ash S, Ward LC, Byrne NM, Green B. 2005. Quantification of lean bodyweight. Clin Pharmacokinet 44:1051–1065.

2. Keizer RJ, Zandvliet AS, Beijnen JH, Schellens JHM, Huitema ADR. 2012. Performance of methods for handling missing categorical covariate data in population pharmacokinetic analyses. AAPS J 14:601–611.
